# Complex effects of the *exo-xis* region of the Shiga toxin-converting bacteriophage Φ24_B_ genome on the phage development and the *Escherichia coli* host physiology

**DOI:** 10.1007/s13353-023-00799-z

**Published:** 2023-11-16

**Authors:** Sylwia Bloch, Bożena Nejman-Faleńczyk, Katarzyna Licznerska, Aleksandra Dydecka, Gracja Topka-Bielecka, Agnieszka Necel, Alicja Węgrzyn, Grzegorz Węgrzyn

**Affiliations:** 1 Department of Molecular Biology, Faculty of Biology, University of Gdansk, Gdansk, Poland; 2 Department of Physiology, Medical University of Gdansk, Gdansk, Poland; 3 Univentum Labs Ltd., Gdansk, Poland; 4 Department of Medical Microbiology, Faculty of Medicine, Medical University of Gdansk, Gdansk, Poland; 5 Phage Therapy Center, University Center for Applied and Interdisciplinary Research, University of Gdansk, Gdansk, Poland

**Keywords:** Shiga toxin-producing *E. coli* (STEC), Stx phages, *exo-xis* region, The lysis vs. lysogenization decision, Gene expression

## Abstract

Lambdoid bacteriophages are excellent models in studies on molecular aspects of virus-host interactions. However, some of them carry genes encoding toxins which are responsible for virulence of pathogenic strains of bacteria. Shiga toxin-converting bacteriophages (Stx phages) encode Shiga toxins that cause virulence of enterohemorrhagic *Escherichia coli* (EHEC), and their effective production depends on Stx prophage induction. The *exo-xis* region of the lambdoid phage genome consists of genes which are dispensable for the phage multiplication under laboratory conditions; however, they might modulate the virus development. Nevertheless, their exact effects on the phage and host physiology remained unclear. Here, we present results of complex studies on the role of the *exo-xis* region of bacteriophage Φ24_B_, one of Stx2b phages. Transcriptomic analyses, together with proteomic and metabolomic studies, provided the basis for understanding the functions of the *exo-xis* region. Genes from this region promoted lytic development of the phage over lysogenization. Moreover, expression of the host genes coding for DnaK, DnaJ, GrpE, and GroELS chaperones was impaired in the cells infected with the Δ*exo-xis* phage mutant, relative to the wild-type virus, corroborating the conclusion about lytic development promotion by the *exo-xis* region. Proteomic and metabolomic analyses indicated also modulation of *gad* and *nrf* operons, and levels of amino acids and acylcarnitines, respectively. In conclusion, the *exo-xis* region controls phage propagation and host metabolism by influencing expression of different phage and bacterial genes, directing the virus to the lytic rather than lysogenic developmental mode.

**Supplementary Information:**

The online version contains supplementary material available at 10.1007/s13353-023-00799-z.

## Introduction

Bacteriophage λ, a virus infecting *Escherichia coli*, has been used as a model in molecular biology for over 60 years. Many crucial discoveries were made during studies on this virus (Casjens and Hendrix 2015). Nevertheless, it still plays its role in molecular biology, and perhaps surprisingly, novel exciting discoveries are being made with λ, including discovery of molecular regulatory mechanisms and development of novel methods in genetic engineering and biotechnology (Caldwell and Bell 2019; Zhao et al. 2023).

While most *Escherichia coli* strains are harmless commensals, the Shiga toxin-producing *E. coli* (STEC) strains and related enterohemorrhagic *E. coli* (EHEC) subtypes contribute to food poisoning and kidney disorders in human populations throughout the world (Kaper et al. 2004; Bugarel et al. 2011). In the USA, STEC causes 100,000 illnesses, 3000 hospitalizations, and 90 deaths annually (Al Qabili et al. 2022), with comparable effects in other countries (Auvray et al. 2023; Matussek et al. 2023; Rodwell et al. 2023; Svendsen et al. 2023).

The main virulence factors of STEC are Shiga toxins (Stx), encoded by genes *stx1* and *stx2*, located in genomes of bacteriophages, which occur in bacteria as prophages (Mizutani et al. 1999). Shiga toxin-converting bacteriophages (Stx phages) belong to the lambdoid family of phages (Besser et al. 1999; Schmidt 2001). All lambdoid phages share many similarities in the life cycle and genome organization. These bacteriophages can undergo either a lytic or lysogenic development (Ptashne 2004; Węgrzyn and Węgrzyn 2005; Kędzierska et al. 2007).

When the lysogenic pathway is chosen, phage DNA is incorporated into *E. coli* genome, forming a prophage that can be maintained in this state for many cell generations. The prophage is replicated together with the bacterial genome and is transmitted to daughter cells at each subsequent cell division. As a result of prophage induction, caused by different factors like low pH, iron ions, antibiotics (Kimmitt et al. 1999, 2000), or hydrogen peroxide (Wagner et al. 2001a; Łoś et al. 2009, 2010), the excision of the phage genome occurs and efficiency of expression of the *stx* genes increases rapidly. Consequently, at this stage, the viral genomes exist as separate DNA molecules in the bacterial cell and replicate separately from the host nucleoid as extrachromosomal elements (Nejman et al. 2009, 2011).

During the lytic development, there are many round of phage DNA replication, and the genes encoding head, tail, and lysis proteins are expressed. This leads to the assembly of many (100–200, on average, under optimal conditions) new phage particles within the cell. The development ends with disruption of the host cell and liberation of progeny virions.

When *E. coli* strain infecting human gut contains an Stx prophage, its induction ends with by the release of Stx progeny phages and Shiga toxin molecules, produced at the time of the expression of phage genes. It is crucial to stress that the effective production of Shiga toxins occurs only upon prophage induction and its further lytic development (Wagner et al. 2001b, 2002; Waldor and Friedman 2005). Treatment of infection caused by STEC bacteria is difficult because many therapeutics, including antibiotics, are prophage inducers. Thus, their application increases expression of toxin genes and enhances the severity of the disease (Kimmitt et al. 2000; Gamage et al. 2004; Serna and Boedeker 2008). In the light of this problem, it is obvious that understanding the mechanisms of regulation of development of lambdoid phages is crucial for both basic knowledge and putative further work on prevention and treatment of STEC infections. Although bacteriophage λ, the best investigated member of lambdoid phage family, has served as a model organism in molecular biology for over 60 years (Węgrzyn et al. 2012), the functions of some of its genes are still unknown.

**Fig. 1:**
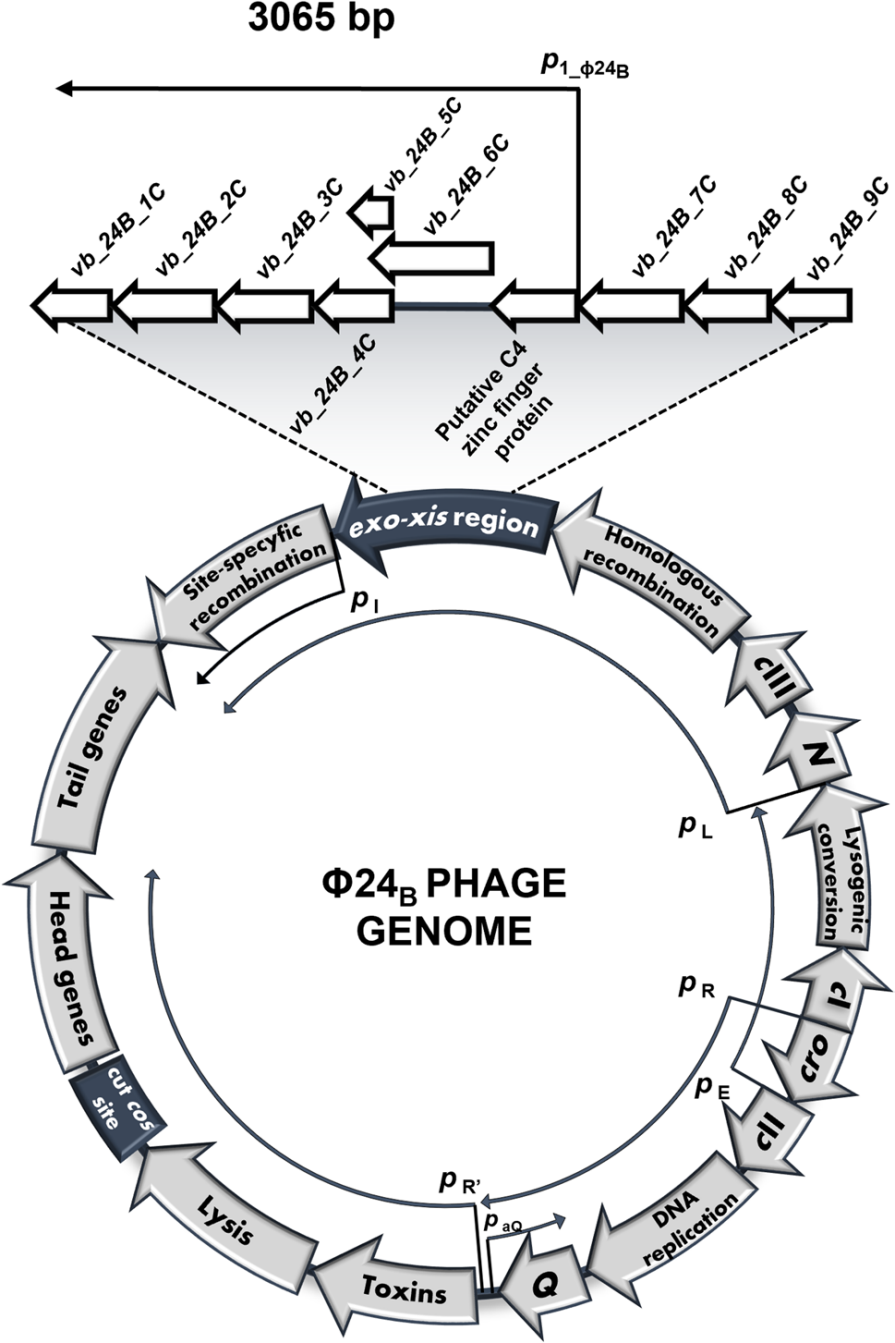
A simplified circular map of bacteriophage Φ24_B_ genome, highlighting phage genes and promoters of the *exo-xis* region. Promoters are depicted by arrows and thin black lines

For many years, functions of the region located between *exo* and *xis* genes of the phage genome (called the *exo-xis* region or the *bin* region) have been almost completely unknown, despite its conservation among lambdoid phages. This region is located in the central part of the phage genome and is transcribed from the early p_L_ promoter which is repressed by the phage-encoded cI protein during lysogeny (Fig. 1). Studies performed by Kourilsky and Knapp (1974) indicated that in the case of bacteriophage λ, transient expression of genes of the p_L_ operon can induce cell-cycle synchrony in a population of host cells. This assumption was confirmed by Sergueev et al. (2002), who showed that induction of the p_L_ operon give rise to two separable effects on the host cell cycle: (i) a temporary block of cell division and, at the same time, (ii) a block of the initiation of DNA replication (hence the term the *bin* region).

Despite the evidence that the *exo-xis* region modulates the host genome function, the physiological significance of this regulation in the bacteriophage development remains unknown. The prevailing hypothesis since 2002 is that *exo-xis* region genes regulate the phage decision whether to lysogenize the host cell or to enter the lytic development (Sergueev et al. 2002). It was speculated that transient inhibition of host DNA replication may help phage, which uses the host replication proteins, to maximize its DNA replication during lytic development (Sergueev et al. 2002). However, no experimental data supporting such a hypothesis were presented until 2008, when the first results presenting an evidence for the relation between *exo-xis* region and phage development were shown (Łoś et al. 2008). These data indicated that the *exo-xis* region contributes to a decreased transcription from cII-stimulated promoters p_I_, p_aQ_, and p_E_. That work displayed also that the *exo-xis* dependent decrease in the efficiency of λ plating on host strain requires the intact *c*I gene. However, the absence of *c*I activity could be compensated by either increased stability of cII or cII-independent transcription of *c*I, which in turn suggests that transcription in this region, but not necessarily the *c*I activity, is responsible for this effect (Łoś et al. 2008). Besides, overproduction of cIII significantly enhanced the negative effect of the *exo-xis* region on λ plating (Łoś et al. 2008).

The *ea8.5* gene, located in the *exo-xis* region, appeared to have the strongest effects on the phage development (Łoś et al. 2008). Other well-known genes involved in the lysis *vs*. lysogenization decision (*c*I, *c*II, and *c*III) were shown to participate in the processes leading to phenotypic effects of the *exo-xis* region. Those results allowed to conclude that the *exo-xis* region may be involved in the regulation of bacteriophage λ development, most probably at the stage of the lysis vs. lysogenization decision; however, it was not enough knowledge to indicate the mechanism of this phenomenon (Łoś et al. 2008).

In the subsequent work, effects of expression of genes from the *exo-xis* region on various stages of development of phages λ and Φ24_B_ (an Stx2b phage) were investigated (Bloch et al. 2013). It was demonstrated that the presence of this region on a multicopy plasmid in naive *E. coli* resulted in impaired lysogenization of host bacteria and more effective induction of prophages, both spontaneous and stimulated by various agents (mitomycin C, H_2_O_2_, and UV radiation) (Bloch et al. 2013). Moreover, patterns of expressions of particular genes from the *exo-xis* region were determined, but unexpectedly, in both phages λ and Ф24_B_, these patterns were significantly different not only between conditions of the host cell infection by bacteriophages and prophage induction, but also between induction of prophages with various agents (mitomycin C and hydrogen peroxide) (Bloch et al. 2014).

Intriguingly, in hydrogen peroxide-treated *E. coli* cells lysogenic for either λ or Φ24_B_, deletion of the *exo-xis* region resulted in a significant decrease in the levels of expression of the S.O.S. regulon genes (Licznerska et al. 2016). Moreover, under these conditions, a dramatic decrease in the levels of expression of phage genes crucial for lytic development (particularly *xis*, *exo*, *N*, *cro*, *O*, *Q*, and *R*) could be observed in Φ24_B_-, but not in λ-bearing cells (Licznerska et al. 2016). That finding indicated that genes located in the *exo-xis* region are necessary for efficient expression of both host S.O.S. regulon in lysogenic bacteria and regulatory genes of Shiga toxin-converting bacteriophage Φ24_B_.

The studies described briefly above indicated intriguing regulatory effects of the *exo-xis* region. However, despite further charactering principles of structures and basic functions of various products of genes from this region, like Ea8.5 (Kwan et al. 2013), Orf63 (Dydecka et al. 2017), Orf60a and Orf61 (Dydecka et al. 2018), Orf73 (Zdrojewska et al. 2019), and Ea22 (Dydecka et al. 2020; Tong et al. 2020), the role of the *exo-xis* region in modulating phage development and influencing host physiology remains unclear. Therefore, the aim of this work was to determine effects of the *exo-xis* region on development of the Stx phage Φ24_B_ and physiology and metabolism of its host. This aim was realized by employing transcriptomic, proteomic, and metabolomic approaches which allow to analyze global changes in gene expression, protein content, and metabolic status in the investigated cells under specific conditions, in this case upon infection with the studied bacteriophages.

## Materials and methods

### Bacterial strains, plasmids, and bacteriophages

*E. coli* MG1655 strain, its derivatives, bacteriophages, and plasmids are presented in Table 1.

**Table 1:** Bacterial strains, bacteriophages, and plasmids used in in  vivo experiments

*E. coli strains, bacteriophages, or plasmids*	Relevant genotype or description	References
*E. coli* laboratory strains		
MG1655	F^–^λ^–^*ilvG rfb-50 rph-1*	Jensen (1993)
MG1655 (Φ24_B_)	MG1655-bearing Φ24_B_ prophage	Bloch et al. (2013)
MG1655 (Φ24_B_Δ*exo-xis*)	MG1655-bearing Φ24_B_ prophage with deletion of the region located between *exo* and *xis genes*	Licznerska et al. (2016)
Bacteriophages		
Φ24_B_	∆s*tx2*::*cat*	Allison et al. (2003)
Φ24_B_Δ*exo-xis*	Φ24_B_ with deletion the region located between *exo* and *xis* genes	Licznerska et al. (2016)
Plasmids		
pUC18	*ori* pMB1 (pBR322 derivative), *bla*, Amp^R^	Thermo Fisher Scientific
pUC18_Φ24_B__*exo-xis*	as pUC18 but bearing the region located between the *exo* and *xis* genes from phage Φ24_B_	This study

The deletion mutant of Φ24_B_ phage, lacking the sequence of the region located between *exo* and *xis* genes, was constructed as described previously (Licznerska et al. 2016), by using *E. coli* MG1655 (Φ24_B_) strain and the Quick and Easy *E. coli* Gene Deletion Kit (Gene Bridges, Heidelberg, Germany). Plasmid pUC18 (Thermo Fisher Scientific Inc., Waltham, MA, USA) was employed as a control variant in all experiments presented in this work. For construction of the plasmid pUC18_*exo-xis*_Φ24_B_, nucleotide sequences of all *orf*s and genes localized between *exo* and *xis* genes from the genome of phage Φ24_B_ were amplified by the PCR method with primers F_Φ24_B__exo-xis_HindIII (5′GTC AAG CTT GAA GGC GGT TGT TAG) and R_Φ24_B__exo-xis_EcoRI (5′TGA GGA TCC GTA TAT GGG GAG CAA TG). Specific oligonucleotide primers were developed by Primer3web version 4.1.0 and synthesized by GENOMED S.A. (Warsaw, Poland). Phage DNA was isolated by using MasterPure™ Complete DNA and RNA Purification Ki (Biosearch Technologies, Hoddesdon, Great Britain). Following digestion with HindIII and BamHI restriction endonucleases (Thermo Fisher Scientific Inc., Waltham, MA, USA), the Φ24_B_ *exo-xis* region was ligated with the HindIII-BamHI fragment of plasmid pUC18 bearing an ampicillin-resistant gene and the *p*_lac_ promoter. Ligation was conducted by using the T4 DNA ligase (Thermo Fisher Scientific Inc., Waltham, MA, USA) according the manufacturer’s protocol. The construction of the pUC18_exo-xis_Φ24 was confirmed by DNA sequencing (GENOMED, Warsaw, Poland).

### Bacterial growth media and cultures

Bacteria were routinely cultured in the Luria-Bertani (LB) medium (BioShop, Burlington, ON, Canada), supplemented with 10 mM CaCl_2_ (Chempur, Piekary Śląskie, Poland) and 10 mM MgSO_4_ (Chempur, Piekary Śląskie, Poland). Where appropriate, the following antibiotics were added: chloramphenicol up to 2.5 μg/ml (BioShop, Burlington, ON, Canada) or ampicillin up to 50 μg/ml (BioShop, Burlington, ON, Canada). The LB broth supplemented with 1.5% agar (LA; BTL, Łódź, Poland) was used as a bottom agar for pouring the Petri dishes. Top agar consisted of LB medium and 0.7% bacteriological agar (BTL, Łódź, Poland). The host bacteria were grown under aeration conditions, achieved by shaking, at 30 °C (most experiments) or 37 °C (preparation of phage lysate). The Petri dishes with bacterial strains were incubated overnight at 37 °C.

### Propagation of bacterial viruses

Bacteria lysogenic with tested bacteriophages, Φ24_B_ or Φ24_B_Δ*exo-xis*, were grown in LB medium to OD_600_ of 0.1. Prophage induction was provoked by addition of mitomycin C (BioShop, Burlington, ON, Canada) to a final concentration of 1 μg/ml. The mixture was incubated at 37 °C with shaking until lysis occurred. The obtained lysate was treated with 4% chloroform for 15 min* (*Chempur, Piekary Śląskie, Poland) and cleared of cellular debris by centrifugation (2000 × *g*, 10 min, 4 °C). In the next step, polyethylene glycol 8000 (PEG8000; BioShop, Burlington, ON, Canada) was added to a final concentration of 10% and stirred overnight at 4 °C. The precipitate was collected by centrifugation (8000 × *g*, 20 min, 4 °C) and suspended in TM buffer (10 mM Tris-HCl, 10 mM MgSO_4_; pH 7.2). To recover virions, the suspension was treated with equal volume of chloroform and centrifuged (2000 × *g*, 10 min, 4 °C). The extraction procedure was repeated until no white interface between the aqueous and organic phases was visible. Finally, the upper phase with phage particles was collected and the titer of the phage lysate was determined by using a plaque assay described below.

### Bacteriophage lysate titration

Bacteriophage titration procedure was performed using standard Petri dishes filled with 25 ml of LA medium. Soft agar (0.7%), supplemented with 10 mM CaCl_2_ and 10 mM MgSO_4_, was maintained at 60 °C before use. The top layer was prepared by mixing 1 ml of an overnight bacterial host culture with 2 ml of the soft agar. The tube with the mixture was slowly rotated for 10–15 s and then immediately poured onto LA bottom agar supplemented with 2.5 μg/ml of chloramphenicol. To determine the number of phage particles per 1 ml of suspension (plaque forming units per ml, PFU/ml), the obtained phage lysate was serially 10-fold diluted in TM buffer (10 mM Tris-HCl, 10 mM MgSO_4_; pH 7.2) and then 2.5 μl of each dilution was spotted on the surface of the top agar. The Petri dishes were incubated at 37 °C for 20 h. The next day, single plaques were counted, and the phage titer was calculated.

### One-step growth experiment

To assess the kinetics of intracellular phage development, one-step growth experiment was performed according to the previously described procedure (Bloch et al. 2014). Briefly, host bacteria were grown in LB medium at 30 °C to an OD_600_ of 0.2. Then, 10 ml of bacterial culture was centrifuged (2000 × *g*, 10 min, 4 °C) and the pellet was suspended in 1 ml of LB medium supplemented with 3 mM sodium azide (Merck, Darmstadt, Germany), 10 mM CaCl_2_, and 10 mM MgSO_4_. Then, phage lysate was added to the sample to multiplicity of infection (m.o.i.) of 0.05 and allowed to adsorb on the bacterial cells surface for 10 min at 30 °C. After incubation, unadsorbed phages were removed by 3-times washing in LB medium with 3 mM sodium azide and centrifugation (2000 × *g*, 10 min, 4 °C). In the next step, 25 μl of the mixture was transferred to the flask filled with 25 ml of fresh, pre-warmed to 30 °C LB medium (time 0). The infected bacterial cultures were aerated in an incubator shaker at 30 °C. The number of infective centers was estimated from nine samples taken in the interval of 0–15 min after the dilution and plating at the permissive conditions. Samples withdrawn at later times were shaken vigorously with equal volume of chloroform and cleared by centrifugation (2000 × *g*, 5 min, RT). The number of intracellular progeny phages was estimated by plating on appropriate indicator *E. coli* MG1655 strain. Plates were incubated overnight at 37 °C and then burst size was estimated as a ratio of phage titer at particular time points to the titer of infection centers.

### Efficiency of lysogenization

To estimate the efficiency of lysogenization, the procedure described earlier (Dydecka et al. 2017) was used, with some modifications. Briefly, host bacteria were cultivated in LB medium with shaking at 30 °C to OD_600_ of 0.2. Then, 1 ml of the sample was centrifuged (2000 × *g*, 10 min, 4 °C) and the bacterial pellet was washed 2 times with TCM buffer (10 mM Tris-HCl, 10 mM MgSO_4_, 10 mM CaCl_2_; pH 7.2). Finally, the obtained pellet was suspended in 1 mL of TCM buffer and phage lysate was added to the mixture to an m.o.i. of 5. After incubation of the sample at 30°, the serial 10-fold dilutions in TCM buffer (10 mM Tris-HCl, 10 mM MgSO_4_, 10 mM CaCl_2_; pH 7.2) were prepared and 20 μl of each dilution was plated on LA plates. Following overnight incubation at 37 °C, 96 bacterial colonies were cultivated in a multi-well plate containing 200 μl of LB. Each plate was shaken at 37 °C until OD_600_ reached 0.1. Then, the putative lysogens were treated with ultraviolet light at 50 J/m^2^ for 20 s and cultivated with shaking for the next 2 h at 37 °C. Afterward, 10 μl of chloroform was added to each well, the plates were centrifugated (2000 × *g*, 10 min, 4 °C) and 2.5 μL of the supernatant was spotted onto double-layer LB agar plates with 2.5 μg/ml of chloramphenicol. Plates were incubated overnight at 37 °C, and then, the efficiency of prophage formation was calculated as a percentage of lysogens among all tested 96 bacterial colonies.

### Survival of host bacteria after phage infection

To determine the survival of host bacteria after phage infection, the procedure described earlier (Dydecka et al. 2017) was used. Briefly, bacteria were grown in LB medium at 30 °C to OD_600_ of 0.2. Sample of 4 ml was centrifuged (2000 × *g*, 10 min, 4 °C) and the obtained bacterial pellet was washed (2000 × *g*, 10 min, 4 °C) twice with cold 0.85% sodium chloride (Chempur, Piekary Śląskie, Poland). Finally, the bacterial pellet was suspended in 1 ml of LB medium supplemented with 10 mM CaCl_2_ and 10 mM MgSO_4_ and then phage lysate was added to an m.o.i. of 5. After incubation at 30 °C, serial dilutions in 0.85% sodium chloride were prepared and 40 μl were spread onto Petri dishes filled with the solid LA medium. Plates were incubated overnight at 37 °C. Percentage of survivors was calculated relative to bacterial sample in which TM buffer was added instead of phage virions.

### Efficiency of phage adsorption

The rate of adsorption of virions on the surface of host cells was determined according to the procedure described previously (Bloch et al. 2013), with some modifications. Briefly, host bacteria were grown in LB medium at 30 °C until OD_600_ reached 0.1. Sample of 1 ml was centrifuged (2000 × *g*, 10 min, 4 °C) and the pellet was washed 2 times with 0.85% sodium chloride (2000 × *g*, 10 min, 4 °C). Finally, the bacterial pellet was suspended in 150 μl of LB medium supplemented with 10 mM CaCl_2_ and 10 mM MgSO_4_. Tested bacteriophages were added to the bacterial samples to an m.o.i. of 0.05, and these mixtures were incubated at 30 °C for 20 min. During incubation, the phage titer was determined at indicated times. Petri dishes were incubated at 37 °C for 18 h. A bacterial sample withdrawn immediately after addition of phage virions to bacterial host suspension (time 0) was considered as 100% of non-adsorbed bacteriophages and other values were calculated relative to them.

### Bacteriophage infection procedure

Infection of host bacteria with tested phages was prepared according to the procedure described previously (Bloch et al. 2014). Briefly, *E. coli* MG1655 was grown in LB medium with aeration at 30 °C to OD_600_ of 0.3. After centrifugation of 120 ml of bacterial culture (2000 × *g*, 10 min, 4 °C), the obtained pellet was suspended in 36 ml of LB medium with 10 mM CaCl_2_ and 10 mM MgSO_4_. Then, phage lysate was added to the sample to an m.o.i. of 5. Following incubation on ice, infected host bacteria were cultivated with shaking at 30 °C. At indicated times, bacterial cells were harvested and treated with the killing buffer (200 mM NaN_3_, 50 mM MgCl_2_, 200 mM Tris-HCl; pH 8.0) to stop their growth. After centrifugation (2000 × *g*, 10 min, 4 °C), bacterial pellet was frozen in liquid nitrogen and then stored at −*80 °C* for further analysis.

### Isolation of total RNA from bacterial cells for microarray analysis and RT-qPCR

For microarray analysis, total RNA was isolated from 7.5 × 10^8^ bacterial cells harvested at 30 min after phage infection. RNA used for RT-qPCR was the same RNA sample as for the microarray analysis; however, to determine the expression patterns of particular genes during the infection process of host bacteria with tested phages, RNA was also extracted from cells at additional time points: 0, 7.5, 15, 45, and 60 min. Ribonucleic acids were isolated using the RNeasy Mini kit (Qiagen, Hilden, Germany). To remove DNA contaminants, the TURBO DNA-free™ Kit (Thermo Fischer Scientific, Waltham, MA, USA) was employed. The quality of the obtained RNA samples was tested with the Nano Chips RNA kit (Agilent Technologies, Santa Clara, CA, USA) using the Agilent 2100 Bioanalyzer System.

### Microarray analysis

Gene expression microarray analyses were carried out commercially by the Agilent Technologies, Inc. (Santa Clara, CA, USA) using Agilent’s *E. coli* Microarray Kit 8x15K, P/N G4813A (Agilent Microarray Design ID 020097). Two-color (Cy3 and Cy5) RNA spike-ins were added to the RNA samples from the Two-Color RNA Spike-In kit (P/N 5188-5279). The labeling and hybridizations of target RNAs were performed by employing the Two-Color (Cy3 and Cy5) Low Input Quick Amp Gene expression kit and the Gene Expression Hybridization kit according to patented protocol of the Agilent Technologies, Inc. Each microarray was composed of 15,208 probes representing the complete genomes of four *E. coli* strains: K-12 MG1655, O157:H7 VT2-Sakai, CFT073, and EDL933 (Guernec et al. 2013). The biological replicates of prepared samples were randomly separated onto two microarray slides. The slides were scanned with the Agilent High Resolution C Scanner (G2656CA) according to the two-color microarray assay scanning protocol (Agilent Technologies, Inc.) and raw microarray image files were generated. Agilent Feature Extraction Image Analysis Software (Version 10.7.3) was involved to extract raw microarray data image files. The microarray data were analyzed for gene expression using the Gene Expression workflow in GeneSpring GX (Version 13.0) Software. Default flag settings were used to make the detection calls. Signal intensities for each probe were normalized to the 75th percentile without baseline transformation. Dye swap arrays were identified for the control vs. treated experiments. The software was used to visualize QC metrics, and the signal values were transferred to SpotFire for correlation analysis. Analysis was carried out an entity list consisting of “detected” probes only. The analysis was carried out using this filtered entity list, using a *t*-test unpaired statistical method with Benjamini Hochberg FDR method. The *p*-values were computed asymptotically. Statistical significance was assessed at FDR < 0.05 and *p* < 0.05.

### Relative RT-qPCR and data analysis

Preparation of cDNAs from 1.25 μg of RNA samples for RT-qPCR was performed with Transcriptor Reverse Transcriptase (Roche Diagnostics International, Rotkreuz, Switzerland) and random hexamer primers (Roche Diagnostics International, Rotkreuz, Switzerland), according to the manufacturer’s instructions. All obtained cDNAs were 10-fold diluted in DEPC-treated water (Thermo Fischer Scientific, Waltham, MA, USA) and tested in RT-qPCR. Gene expression patterns were determined by RT-qPCR as described previously (Bloch et al. 2014). All experiments were performed by using the LightCycler® 480 Real-Time PCR System (Roche Diagnostics International, Rotkreuz, Switzerland). Each reaction mixture consisted of 2× SYBR Green I Master Mix, 6.25 ng/μl of cDNA, and 200-nM specific oligonucleotide primers. Amplification was performed according to following program: 95 °C for 5 min, 55 cycles of 95 °C for 10 s, 60 °C for 15 s, and 72 °C for 15 s. All primers (Table 2), amplifying 100–150 nt of target genes, were designed by Primer3web version 4.1.0 and synthetized by GENOMED S.A. (Warsaw, Poland). To confirm the specificity of created primers, melting curve for each product was analyzed. The housekeeping gene *icdA* was employed as a reference for normalization of samples. The samples harvested before addition of phage particles (time point 0) were applied as a calibrator. The relative changes in the level of gene expression were determined with LinRegPCR using the E-Method with efficiency correction (Bloch et al. 2014).

**Table 2:** Oligonucleotide primers used for RT-qPCR

Name	Sequence (5′→3′)
Primers for phage sequences	
pF_Φ24B_exo pR_Φ24B_exo	TGCCGTCACTGCATAAACC TCTATCGCGACGAAAGTATGC
pF_Φ24B_bet pR_Φ24B_bet	GAGACGGGCACACTGAATCA CCGCGAACCATTCAAAACCC
pF_Φ24B_stk pR_Φ24B_stk	CGACGATCAGCAATGCGATG GCATGGATTCTGTCGACCCA
pF_Φ24B_N pR_Φ24B_N	AGGCGTTTCGTGAGTACCTT TTACACCGCCCTACTCTAAGC
pF_Φ24B_cI pR_Φ24B_cI	TGCTGTCTCCTTTCACACGA GCGATGGGTGGCTCAAAATT
pF_Φ24B_cro pR_Φ24B_cro	CGAAGGCTTGTGGAGTTAGC GTCTTAGGGAGGAAGCCGTT
pF_Φ24B_cII pR_Φ24B_cII	TGATCGCGCAGAAACTGATTTAC GACAGCCAATCATCTTTGCCA
pF_Φ24B_O pR_Φ24B_O	AAGCGAGTTTGCCACGAT GAACCCGAACTGCTTACCG
pF_Φ24B_Q pR_Φ24B_Q	GGGAGTGAGGCTTGAGATGG TACAGAGGTTCTCCCTCCCG
pF_Φ24B_R pR_Φ24B_R	GGGTGGATGGTAAGCCTGT TAACCCGGTCGCATTTTTC
pF_Φ24B_P pR_Φ24B_P	TCGTAAAGCGCTACGAGGTT AGTGCTGATTCGCTTGACCT
Primers for bacterial sequences	
pF_Φ24B_dnaK pR_Φ24B_dnaK	CATTGACCTGCGCAACGATC AGGTTAACGTCGGTCTGCTG
pF_Φ24B_dnaJ pR_Φ24B_dnaJ	CAAACGCCTGGCCATGAAAT GCGAGTCGGTCAGAACTTCA
pF_Φ24B_degP pR_Φ24B_degP	CTCGAAAAGGTGATGCCTTC TCATCACCGAAGAACTGCTG
pF_Φ24B_lon pR_Φ24B_lon	GCTGTTCCTGCTCGATGAGA CGCTACGTTCTGCTCTGGAT
pF_Φ24B_clpB pR_Φ24B_clpB	GGCCGAGGAACAGGAATGAA ATTGGTCAGAACGAAGCGGT
pF_Φ24B_grpE pR_Φ24B_grpE	AGGGAAGCACTCACATTGTCA TGTGTTGTCAGTGCAGTGGG
pF_Φ24B_ibpB pR_Φ24B_ibpB	GCGCGTACCTTCCAGTTGAA TTCCCGCCGTACAACATTGA
pF_Φ24B_groL pR_Φ24B_groL	CGCAGCAACCGAAGAATACG TTCGGTGGTGATCATCAGGC
pF_Φ24B_gadC pR_Φ24B_gadC	CCCTGCGGAGAGGTTGATTT CGCGACTATCCGTTGGCTAT
pF_Φ24B_hdeD pR_Φ24B_hdeD	CTCGTTATTGGTGTGCTGGA GCGGCGCTAAATATCAGTTC
pF_Φ24B_gadE pR_Φ24B_gadE	TTGGCACCTTATCACATCAGT TCTTATGGGGCAAGTGTTTACCA
pF_Φ24B_nrfA pR_Φ24B_nrfA	CCCGCAAAACCTGTAACTGT CGTCAACACGCTCTGACTGT
pF_Φ24B_nrfD pR_Φ24B_nrfD	CTGACAAGACCGTGGACCTT GCACCACCATGTAGAGCTGA

### Proteome analysis using two-dimensional electrophoresis (2D-PAGE)

Samples for proteomic analyses were prepared according to the manual provided by the Kendrick Laboratories (Madison, USA). Following 30-min incubation at 30 °C with tested bacteriophages, 5 × 10^10^ bacterial cells were harvested by centrifugation (4500 × *g*, 5 min, 4 °C). Then, bacterial pellet was deeply frozen in liquid nitrogen and washed 2 times (4500 × *g*, 5 min, 4 °C) with a buffer containing 10 mM Tris-HCl, pH 8.0 (Merck, Darmstadt, Germany), 1 mM KH_2_PO_4_ (Avantor Performance Materials Poland S.A., Gliwice, Poland), 68 mM NaCl (Chempur, Piekary Śląskie, Poland), and 9 mM NaH_2_PO_4_ (Avantor Performance Materials Poland S.A., Gliwice, Poland). Finally, the obtained bacterial pellet was resuspended in 1 ml of a buffer containing 10 mM Tris-HCl, pH 8.0, 1.5 mM MgCl_2_, 10 mM KCl (Chempur, Piekary Śląskie, Poland), 0.2% SDS (Merck, Darmstadt, Germany), supplemented with 1× Halt Protease Inhibitor Cocktail, EDTA-Free (Thermo Fisher Scientific Inc., Waltham, MA, USA). Cells were then disrupted by ultrasonication for 10 min in Omni-Ruptor 4000 apparatus (OMNI International, Kennesaw, USA), in an ice bath. In the next step, the mixture was incubated at 4 °C for 15 min with DNase (Merck, Darmstadt, Germany) and RNase (Merck, Darmstadt, Germany), added to the final concentration of 100 μg/ml and 50 μg/ml, respectively. Sample was diluted in the proportion 1:1 in boiling buffer containing 5% SDS (Merck, Darmstadt, Germany), 10% glycerol, and 60 mM Tris-HCl, pH 6.8, and then incubated at 95 °C for 10 min. The soluble protein fraction was separated from cell remnants by centrifugation (20,000 × *g*, 30 min, 20 °C). Afterward, the concentration of proteins was determined by using the Pierce™ BCA Protein Assay kit (Thermo Fisher Scientific Inc., Waltham, MA, USA) as recommended by the manufacturer. All prepared samples were deeply frozen in liquid nitrogen and analyzed commercially by the Kendrick Laboratories (Madison, USA).

Proteins that were separated by SDS-PAGE/2D-PAGE and stained by Coomassie dye were excised, washed, and the proteins from the gel were treated according to previously published protocols (Shevchenko et al. 1996; Darie et al. 2011; Sokolowska et al. 2012). Briefly, the gel pieces were washed in high-purity, high-performance liquid chromatography HPLC-grade water; dehydrated and cut into small pieces; and destained by incubating in 50 mM ammonium bicarbonate, 50 mM ammonium bicarbonate/50% acetonitrile, and 100% acetonitrile under moderate shaking, followed by drying in a speed-vac concentrator. The gel bands were then rehydrated with 50 mM ammonium bicarbonate. The procedure was repeated twice. The gel bands were then rehydrated in 50 mM ammonium bicarbonate containing 10 mM DTT and incubated at 56 °C for 45 min. The DTT solution was then replaced by 50 mM ammonium bicarbonate containing 100 mM iodoacetamide for 45 min in the dark, with occasional vortexing. The gel pieces were then re-incubated in 50 mM ammonium bicarbonate/50% acetonitrile, and 100% acetonitrile under moderate shaking, followed by drying in speed-vac concentrator. The dry gel pieces were then rehydrated using 50 mM ammonium bicarbonate containing 10 ng/μl trypsin, and incubated overnight at 37 °C with low-intensity shaking. The resulting peptides were extracted twice with 5% formic acid/50 mM ammonium bicarbonate/50% acetonitrile and once with 100% acetonitrile under moderate shaking. Peptide mixture was then dried in a speed-vac, solubilized in 20 μl of 0.1% formic acid/2% acetonitrile.

The peptide mixture was analyzed by reversed phase nanoliquid chromatography (LC) and MS (LC-MS/MS) using a NanoAcuity UPLC (Micromass/Waters, Milford, MA) coupled to a Q-TOF Xevo G2 mass spectrometer (Micromass/Waters, Milford, MA), according to published procedures (Shevchenko et al. 1996; Darie et al. 2011; Sokolowska et al. 2012). Briefly, the peptides were loaded onto a 100 μm × 10 mm NanoAquity BEH130 C18 1.7-μm UPLC column (Waters, Milford, MA) and eluted over a 60-min gradient of 2–80% organic solvent (ACN containing 0.1% FA) at a flow rate of 400 nl/min. The aqueous solvent was 0.1% FA in HPLC water. The column was coupled to a Picotip Emitter Silicatip nanoelectrospray needle (New Objective, Woburn, MA). MS data acquisition involved survey MS scans and automatic data-dependent analysis (DDA) of the top six ions with the highest-intensity ions with the charge of 2+, 3+, or 4+ ions. The MS/MS was triggered when the MS signal intensity exceeded 250 counts/s. In survey MS scans, the three most intense peaks were selected for collision-induced dissociation (CID) and fragmented until the total MS/MS ion counts reached 10,000 or for up to 6 s each. The entire procedure used was previously described (Shevchenko et al. 1996; Darie et al. 2011; Sokolowska et al. 2012). Calibration was performed for both precursor and product ions using 1-pmol GluFib (Glu1-Fibrinopeptide B) standard peptide with the sequence EGVNDNEEGFFSAR and the monoisotopic doubly-charged peak with m/z of 785.84. The raw data were processed using ProteinLynx Global Server (PLGS, version 2.4) software. The following parameters were used: background subtraction of polynomial order 5 adaptive with a threshold of 30%, two smoothings with a window of three channels in Savitzky-Golay mode, and centroid calculation of top 80% of peaks based on a minimum peak width of 4 channels at half height. The resulting pkl files were submitted for database search and protein identification to the in-house Mascot server (http://www.matrixscience.com, Matrix Science, London, UK) for database search using the following parameters: databases from NCBI (bacteria and viruses), parent mass error of 0.5 Da with 1 ^13^C, product ion error of 0.8 Da, and enzyme used: trypsin, three missed cleavages, propionamide as cysteine fixed modification, and methionine oxidized as variable modification. To identify the false negative results, we used additional parameters such as different databases or organisms, a narrower error window for the parent mass error (1.2 and then 0.2 Da) and for the product ion error (0.6 Da), and up to two missed cleavage sites for trypsin. In addition, the pkl files were also searched against in-house PLGS database version 2.4 (http://www.waters.com) using searching parameters similar to the ones used for Mascot search. The Mascot and PLGS database search provided a list of proteins for each gel band. To eliminate false positive results, for the proteins identified by either one peptide or a mascot score lower than 25, we verified the MS/MS spectra that led to identification of a protein. Additionally, image analysis was performed with the use of the DECODON Delta 2D software, version 4.0 (DECODON), which is based on the dual-channel image analysis technique (Bernhardt et al. 1999).

### Extraction and quantification of endogenous metabolites

Samples for endogenous metabolite analyses were prepared according to the procedure described for bacteriophage infection. Following 30-min incubation at 30 °C with tested bacteriophage, 1 × 10^9^ bacterial cells were harvested by centrifugation (2000 × *g*, 10 min, 4 °C). To release metabolites from *E. coli* cells, the cold methanol extraction method (Maharjan and Ferenci 2003) was used with some modifications. Briefly, the obtained pellet was suspended in 1 ml of 50% cold (−20 °C) methanol (Merck, Darmstadt, Germany). After rapid mixing, the sample was frozen in liquid nitrogen, and then thawed on a dry ice. The freeze-thaw cycle was performed 3 times in order to permeabilize the cells, and then the mixture was centrifuged (16,000 × *g*, 10 min, 4 °C). The supernatant was transferred to a new tube and stored in dry ice. The pellet was suspended in 0.5 ml of 50% cold (−20 °C) methanol, intensively mixed, frozen in liquid nitrogen, and then thawed on a dry ice. The procedure of freeze-thawing was repeated 3 times. Following the centrifugation (16,000 × *g*, 10 min, 4 °C), the first and second extracts were combined in a new tube. Finally, the samples were concentrated for 4–6 h in the vacuum centrifugation at 30 °C to approximately 40 μl. Finally, samples from three biological replicates were pooled into two groups.

Absolute *IDQ*® p180 kit plates (Biocrates Life Science, Innsbruck, Austria) were used for the quantification of amino acids, biogenic amines, acylcarnitines, and hexoses in prepared samples. The analysis has been carried out commercially by Biocrates Life Science (Innsbruck, Austria). The fully automated assay was based on phenylisothiocyanate (PITC) derivatization in the presence of internal standards, followed by flow injection analysis-tandem mass spectrometry (FIA-MS/MS; for the analysis of acylcarnitines and hexoses) and liquid chromatography-tandem mass spectrometry (LC-MS/MS; for the analysis of amino acids and biogenic amines) using 4000 QTRAP® (AB Sciex, Darmstadt, Germany) and a Xevo TQ-S micro (Waters, Vienna, Austria) instrument with an electrospray ionization (ESI) source. The experimental metabolomic measurement technique was described previously (Ramsay et al. 2005a, 2005b). Data were quantified using a mass spectrometry software (Sciex Analyst® and Waters MassLynx™) and imported into Biocrates Met*IDQ*™ software for further analysis.

### Statistical analyses

Each experiment was repeated 3 or 4 times. Variation among biological replicates was presented as error bars indicating the standard deviation (SD). Comparison of two average values was performed by using Student’s *t*-test and the significance differences were marked by asterisks as follows: *p* < 0.05 (*), *p* < 0.01 (**), or *p* < 0.001 (***).

### Data availability

Row data of transcriptomic (microarray), proteomic, and metabolomic analyses are presented in the supplementary materials, as Table S1, Table S2, and Table S3, respectively.

## Results

### Effects of the deletion of the exo-xis region on bacteriophage development of the Φ24_B_ phage

**Fig. 2:**
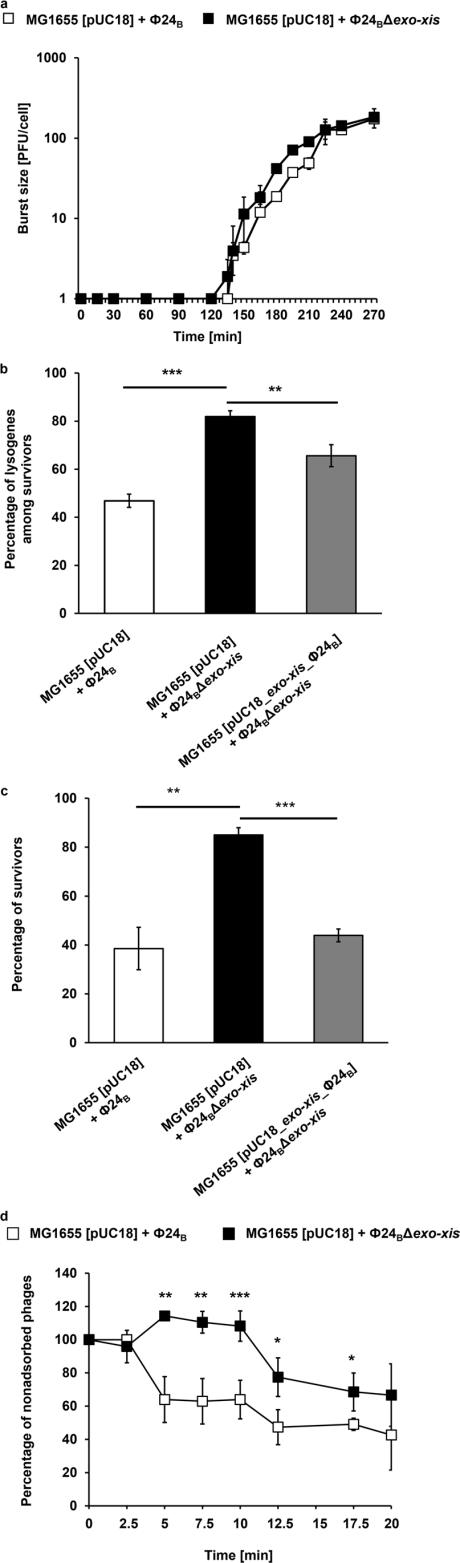
Effects of the *exo-xis* region on bacteriophage Φ24_B_ development. **a** Intracellular development of Φ24_B_ (open squares) and Φ24_B_Δ*exo-xis* (closed squares) bacteriophages after infection of *E. coli* MG1655 [pUC18] at an m.o.i. of 0.05. The presented results are mean values ± SD from three independent experiments. Results are shown as PFU (plaque forming units) per cell. **b** Efficiency of lyzogenization after infection of *E. coli* MG1655 strain bearing pUC18 or pUC18_*exo-xis_*Φ24_B_ plasmid with Φ24_B_ or Φ24_B_Δ*exo-xis* bacteriophage*.* The experiment was conducted in the TCM buffer at 30 °C and at m.o.i. of 5. **c** Survival of host bacteria (%) after infection of *E. coli* MG1655 strain bearing pUC18 or pUC18_*exo-xis_*Φ24_B_ plasmid with Φ24_B_ or Φ24_B_Δ*exo-xis* bacteriophage*.* The experiment was conducted in the LB medium at 30 °C and an m.o.i. of 5. **d** Rate of adsorption of Φ24_B_ (open squares) and Φ24_B_Δ*exo-xis* (closed squares) bacteriophages on *E. coli* MG1655 bacteria bearing pUC18 plasmid. The experiment was conducted in the LB medium at 30 °C and an m.o.i. of 0.05. In panels b, c, and d, the results are presented as mean values ± SD from three independent biological experiments. Statistical analysis was performed by using Student’s *t*-test. The significance of differences between compared experimental variants was marked by asterisks: *p* < 0.01 (**) or *p* < 0.001 (***)

Since most of previously published studies on the role of the *exo-xis* region of the Stx phage Φ24_B_ were conducted in the experimental system with overexpression of the genes from this region (Bloch et al. 2013, 2014), our first attempt was to determine effects of the deletion of this region on the phage development. When comparing the lytic propagation of Φ24_B_ and Φ24_B_Δ*exo-xis* (Fig. 1) phages, no significant differences could be observed (Fig. 2a). However, lysogenization efficiency was significantly more effective in the absence of the *exo-xis* region in the phage genome (Fig. 2b). This was corroborated by the higher fraction of bacteria surviving bacteriophage infection when the Δ*exo-xis* mutant of Φ24_B_ was used (Fig. 2c). The effects of the absence of the *exo-xis* region on the lysogenization or host survival efficiencies could be significantly reduced or completely reversed, respectively, by the presence of this phage genome region in a plasmid in the host cell (Fig. 2b, c). This finding ensured that the observed changes were specific to the *exo-xis* region rather than arising from any polar or side effects. Thus, we confirmed that the *exo-xis* region plays a role in directing the phage Φ24_B_ development into the lytic, rather than lysogenic pathway.

Intriguingly, the kinetics of adsorption of the Φ24_B_Δ*exo-xis* phages on the host cells was significantly slower than that of the wild-type Φ24_B_ phage, though finally, similar efficiency of adsorption was reached by both bacteriophages after 20 min (Fig. 2d). This suggests that expression of the genes located between *exo* and *xis* might influence the structure of the phage virion, especially structures involved in the interactions with the host cell receptor.

### Transcriptomic analyses of the *exo*-*xis* effects on expression of phage and host genes

To learn more about the global effects of the *exo-xis* region on expression of phage and host genes, we have employed transcriptomics. Host cells were infected with either Φ24_B_ or Φ24_B_Δ*exo-xis* and transcriptomes were compared at different times after infection. Both phage and bacterial genes were analyzed.

**Fig. 3:**
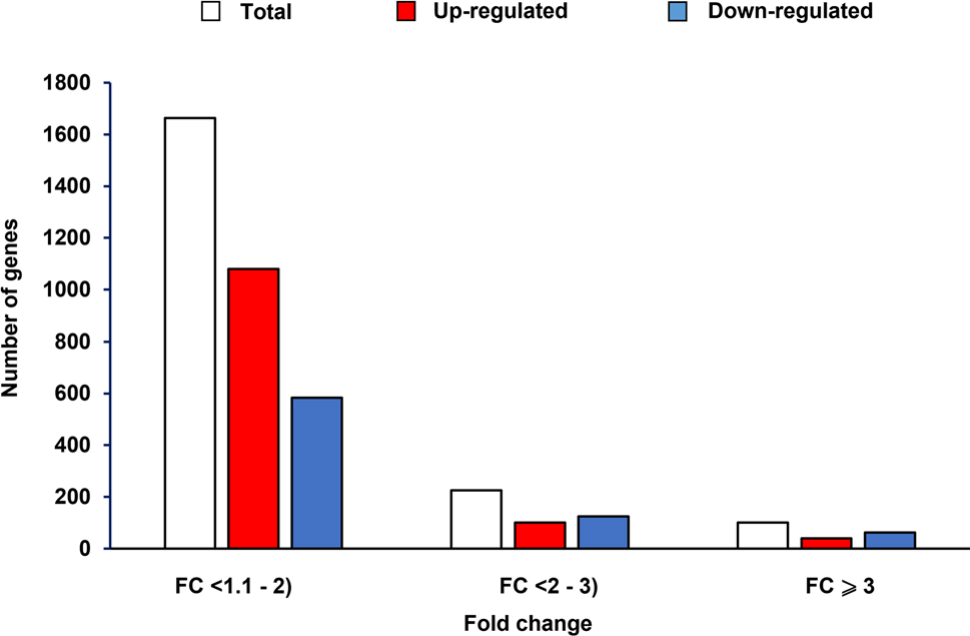
Bacterial and phage genes which expression (measured as transcript levels) was significantly changed (FDR < 0.05 and *p* < 0.05) in *E. coli* MG1655 infected with phage Φ24_B_Δ*exo-xis* relative to host bacteria infected with wild-type phage Φ24_B_, as revealed by transcriptomic (microarray) analysis at time 30 min after infection

**Fig. 4:**
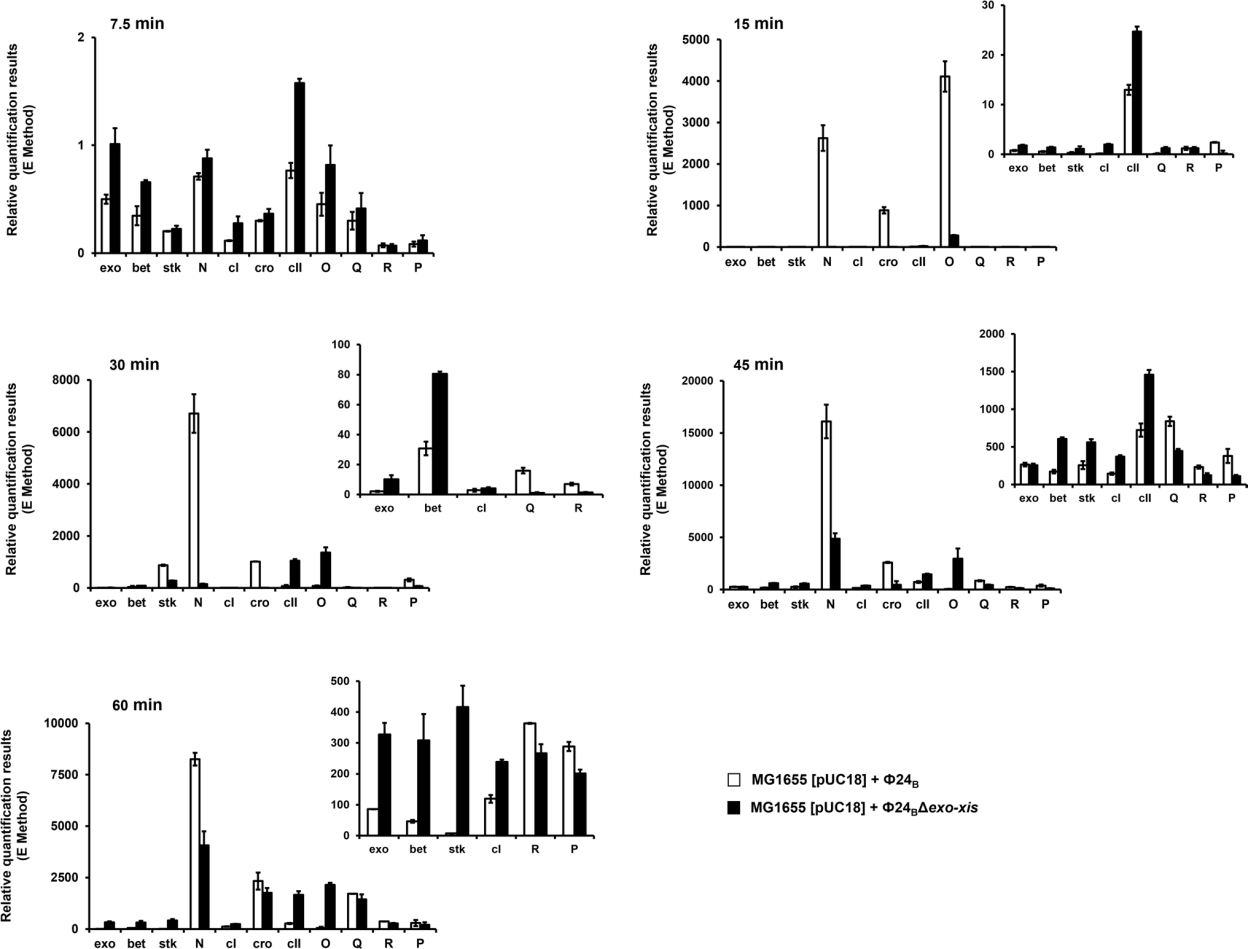
Expression patterns of selected genes of phage Φ24_B_ (open columns) and Φ24_B_∆*exo-xis* (closed columns) infecting *E. coli* MG1655 host at 30 °C, assessed by RT-qPCR analysis. Levels of transcripts corresponding to particular genes were determined at following times after infection: 7.5, 15, 30, 45, or 60 min. The presented results are mean values from three independent experiments with error bars indicating SD

Generally, differences between *E. coli* cells infected with the wild-type phage and that devoid of the *exo-xis* region involved several hundred genes which were differentially expressed (Fig. 3 and Table S1). Abundance of selected transcripts, revealing significant differences between *E. coli* cells infected with Φ24_B_ or Φ24_B_Δ*exo-xis*, was confirmed by using an independent method, RT-qPCR. When analyzing bacteriophage genes, it was evident that those supporting lysogenization, especially *c*I and *c*II, were up-regulated in Φ24_B_Δ*exo-xis*-infected *E. coli* cells relative to the wild-type phage-infected bacteria (Fig. 4). This was true throughout the infection period. Contrary to these genes, expression of the *N* gene, coding for one of major regulators promoting lytic development, was down-regulated in the case of the Φ24_B_Δ*exo-xis* phage (Fig. 4). These results suggest the mechanism of the *exo-xis* region-mediated promotion of the lytic development, namely, negative control of the lysogenization-specific genes and positive control of the expression of those involved in the lytic development. Summary of the transcriptomic analyses of the phage genes is shown in Table 3.

**Table 3:** Bacteriophage genes differentially expressed after infection of *E. coli* MG1655 with Φ24_B_Δ*exo-xis* phage relative to *E. coli* MG1655 infected with Φ24_B_ phage at an m.o.i. of 5, as revealed by the transcriptomic analysis

Gene	Function	Log_2_ FC	*p-*FDR
933W bacteriophage			
*ST933Wp22*	Similar to eukaryotic serine/threonine protein kinases	−1.434	3.0E−05
*ST933Wp23*	RuvC-like Holliday junction resolvase	−1.261	6.0E−03
*ST933Wp24*	DUF3024 domain-containing protein	−1.190	2.7E−03
*ST933Wp25*	Putative repressor protein CI	−1.372	2.3E−03
*ST933Wp47*	Putative endopeptidase Rz	−0.590	1.4E−03
*ST933Wp49*	Putative Bor protein precursor	−0.739	1.5E−02
*ST933Wp59*	Putative tail fiber protein	−1.004	5.5E−04
*ST933Wp65*	Membrane-associated protein	−1.903	1.3E−04
CP-933H bacteriophage			
*Z0310*	Putative CII antiterminator protein	+4.000	1.3E−06
*Z0311*	Partial O replication protein	+4.000	3.8E−06
CP-933K bacteriophage			
*Z0951*	Putative exonuclease	+1.894	1.3E−05
*Z0952*	Putative Bet recombination protein	+1.210	1.2E−04
*Z0977*	Putative tail component	+0.738	1.5E−03
CP-933N bacteriophage			
*Z1764*	Partial integrase	+0.625	3.6E−02
CP-933X bacteriophage			
*Z1874*	Putative antiterminator Q	+0.997	1.9E−02
*Z1878*	Putative Bor protein	−0.585	2.0E−02
CP-933R bacteriophage			
*Z2374*	Putative holin protein of prophage	−0.560	2.3E−02
CP-933V bacteriophage			
*Z3356*	Putative DNA replication protein O	+2.490	7.3E−03
*Z3366*	Putative recombination protein Bet	+1.157	1.2E−04
*Z3367*	Putative exonuclease	+2.062	4.3E−06
CP-933P bacteriophage			
*Z6061*	Putative endonuclease	+0.783	2.8E−02

**Fig. 5:**
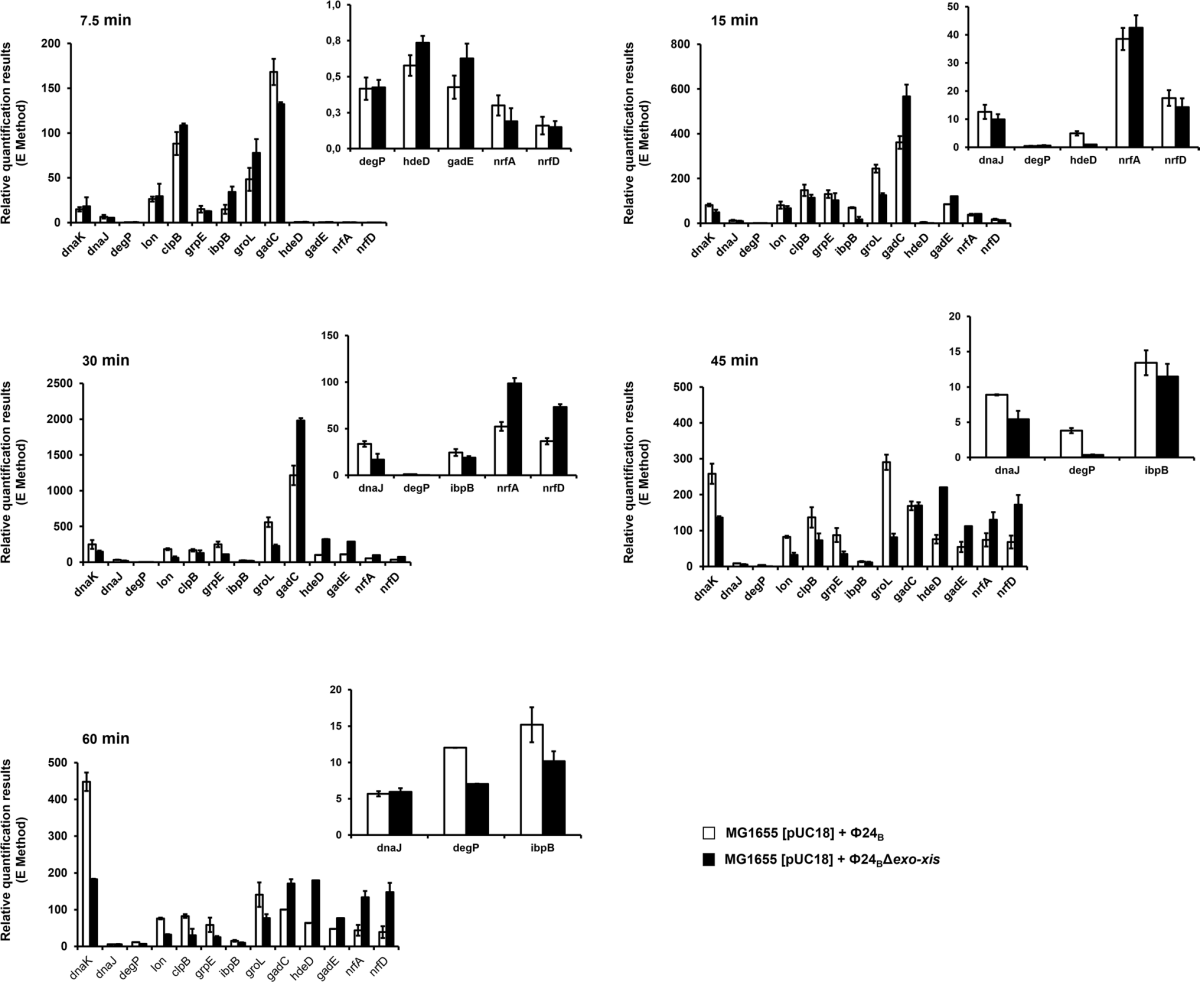
Expression patterns of selected bacterial genes after infection of the *E. coli* MG1655 host with Φ24_B_ (open columns) and Φ24_B_∆*exo-xis* (closed columns) bacteriophages 30 °C, assessed by RT-qPCR analysis. Levels of transcripts corresponding to particular genes were determined at following times after infection: 7.5, 15, 30, 45, or 60 min. The presented results are mean values from three independent experiments with error bars indicating SD

When analyzing transcripts of bacterial genes after infection with Φ24_B_ or Φ24_B_Δ*exo-xis*, it was interesting that those coding for chaperone proteins (DnaK, DnaJ, GrpE, GroELS) were down-regulated in the absence of the *exo-xis* region (Fig. 5). Since chaperone proteins are required for replication of lambdoid phage DNA and assembly of the progeny virions (Węgrzyn et al. 2012), these results suggest another mechanism by which the *exo-xis* region may stimulate lytic development, namely, supporting expression of the molecular chaperone genes. Interestingly, other host genes modulated by the *exo-xis* region were those from the *gad* and *nrf* operons. They are involved in the survival of cells under low pH and anaerobic conditions, respectively. These functions are especially important for bacteriophages which lysogenize bacterial cells, as being prophages they are totally dependent on the host survival under various conditions. A summary of the transcriptomic analyses of host genes after infection with Φ24_B_ or Φ24_B_Δ*exo-xis* is shown in Table 4.

**Table 4:** Bacterial genes differentially expressed after infection of *E. coli* MG1655 with Φ24_B_Δ*exo-xis* phage relative to *E. coli* MG1655 infected with Φ24_B_ phage at an m.o.i. of 5

Gene	Function	Log_2_ FC	*p-*FDR
Chaperones genes			
*vdnaK*	Chaperone protein DnaK	−1.299	1.3E−05
*gggdnaJ*	Chaperone protein DnaJ	−0.889	3.6E−04
*degP*	Periplasmic serine endoprotease DegP	−3.013	1.1E−07
*lon*	Lon protease	−0.960	1.8E−03
*htpX*	Protease HtpX	−2.442	1.3E−06
*cspI*	Cold shock-like protein CspI	−1.737	2.0E−03
*mdtJ*	Multidrug/spermidine efflux pump membrane subunit MdtJ	−1.483	4.2E−04
*htpX*	Protease HtpX	−2.442	1.3E−06
*yehC*	Putative fimbrial chaperone YehC	−1.270	3.5E−04
*lpxP*	Palmitoleoyl acyltransferase	−0.732	1.1E−02
*clpB*	Chaperone protein ClpB	−1.049	1.0E−04
*grpE*	Nucleotide exchange factor GrpE	−1.187	2.4E−05
*ibpB*	Small heat shock protein IbpB	−2.085	5.4E−05
*mdtI*	Multidrug/spermidine efflux pump membrane subunit MdtI	−1.596	4.8E−04
*hslU*	Heat shock protein HslU/ATPase component of the heat shock protein HslU	−1.040	1.1E−04
*hslV*	Heat shock protein HslU/peptidase component of the HslVU protease	−1.091	1.2E−04
*ibpA*	Small heat shock protein Ibp	−1.383	8.7E−06
*groS*	Co-chaperonin GroES	−1.546	2.9E−05
*groL*	Chaperonin GroEL	−0.734	5.1E−03
Genes of acid stress response			
*gadB*	Glutamate decarboxylase B	+1.465	7.7E−03
*gadC*	L-glutamate:4-aminobutyrate antiporter	+1.241	6.5E−03
*asr*	Acid shock-inducible periplasmic protein	+1.557	3.2E−05
*hdeB*	Periplasmic acid stress chaperone HdeB	+1.307	8.0E−04
*hdeA*	Periplasmic acid stress chaperone HdeA	+1.115	3.0E−03
*hdeD*	Acid-resistance membrane protein	+1.441	8.3E−04
*gadE*	DNA-binding transcriptional activator GadE	+1.114	1.2E−03
*gadW*	DNA-binding transcriptional dual-regulator GadW	+1.443	1.8E−04
*gadX*	DNA-binding transcriptional dual-regulator GadX	+1.603	1.5E−04
*gadA*	Glutamate decarboxylase A	+1.629	1.7E−03
*nrf* operon			
*nrfA*	Cytochrome c552 nitrite reductase	+1.422	1.22E−04
*nrfB*	Periplasmic nitrite reductase penta-heme c-type cytochrome	+1.115	2.59E−03
*nrfC*	Putative menaquinol-cytochrome C reductase 4Fe-4S subunit	+1.345	1.37E−03
*nrfD*	Putative menaquinol-cytochrome C reductase subunit NrfD	+1.301	1.52E−03
*nrfE*	Putative cytochrome C-type biogenesis protein NrfE	+0.597	1.37E−02
*nrfF*	Putative formate-dependent nitrite reductase complex subunit NrfF	+0.845	8.54E−03
*nrfG*	Putative formate-dependent nitrite reductase complex subunit NrfG	+0.768	1.51E−03

**Fig. 6:**
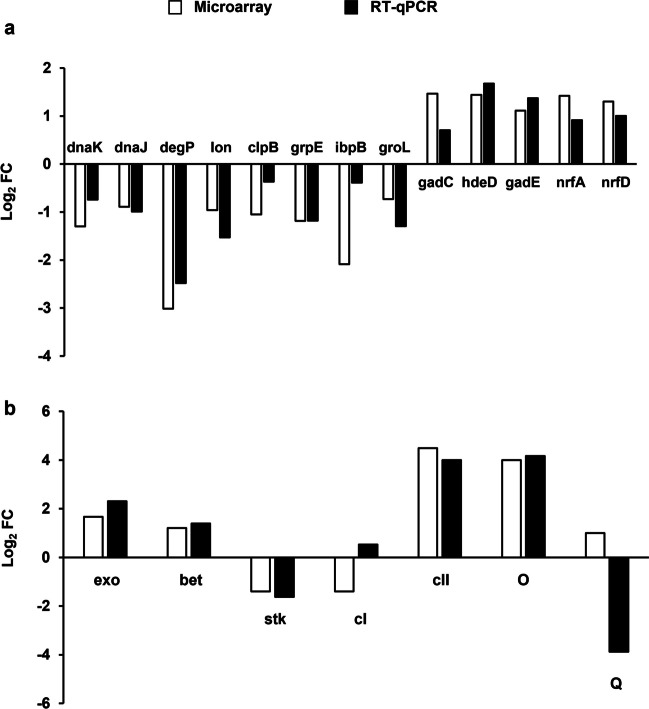
Comparison of changes in levels of selected bacterial (**a**) and phage (**b**) transcripts in *E. coli* MG1655 infected with phage Φ24_B_Δ*exo-xis*, assessed by the microarray technique (open columns) and the RT-qPCR (closed columns) method, relative to host bacteria infected with wild-type phage Φ24_B_ at time 30 min after infection

As mentioned above, among several hundred genes whose expressions were changed as revealed by the microarray analysis (Fig. 3), some were selected for more detailed analysis (Figs. 4 and 5) on the basis of the specific fold-change values and potential importance for bacteriophage development and host survival. To estimate general reliability of the transcriptomic data and validity of the choice of the genes for more detailed studies, we have confirmed that results of the gene expression microarray analyses were compatible with those obtained in reverse transcriptase quantitative PCR (RT-qPCR) experiments (Fig. 6). This indicated reliability of the analyzed transcriptomic results.

### Proteomic analyses of the *exo*-*xis* effects on levels of proteins in host cells

**Fig. 7:**
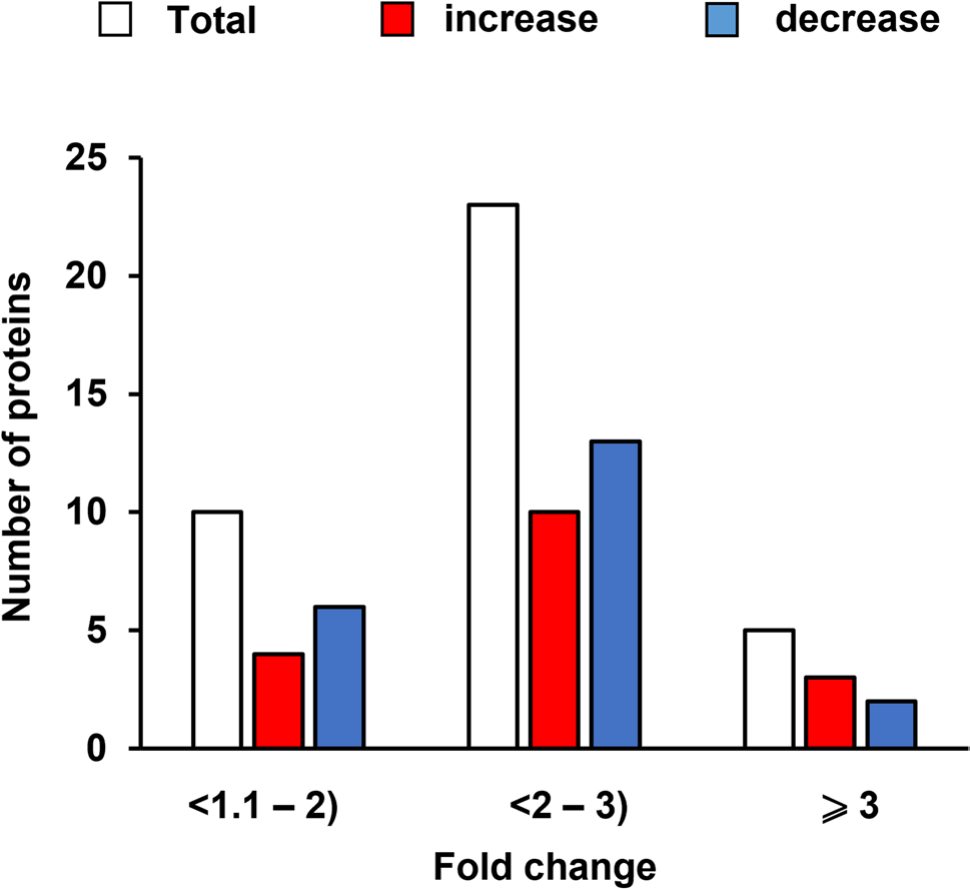
Number of proteins which levels were significantly increased (red columns) or decreased (blue columns) in *E. coli* MG1655 infected with phage Φ24_B_Δ*exo-xis* relative to host bacteria infected with wild-type phage Φ24_B_ at time 30 min after infection

**Fig. 8:**
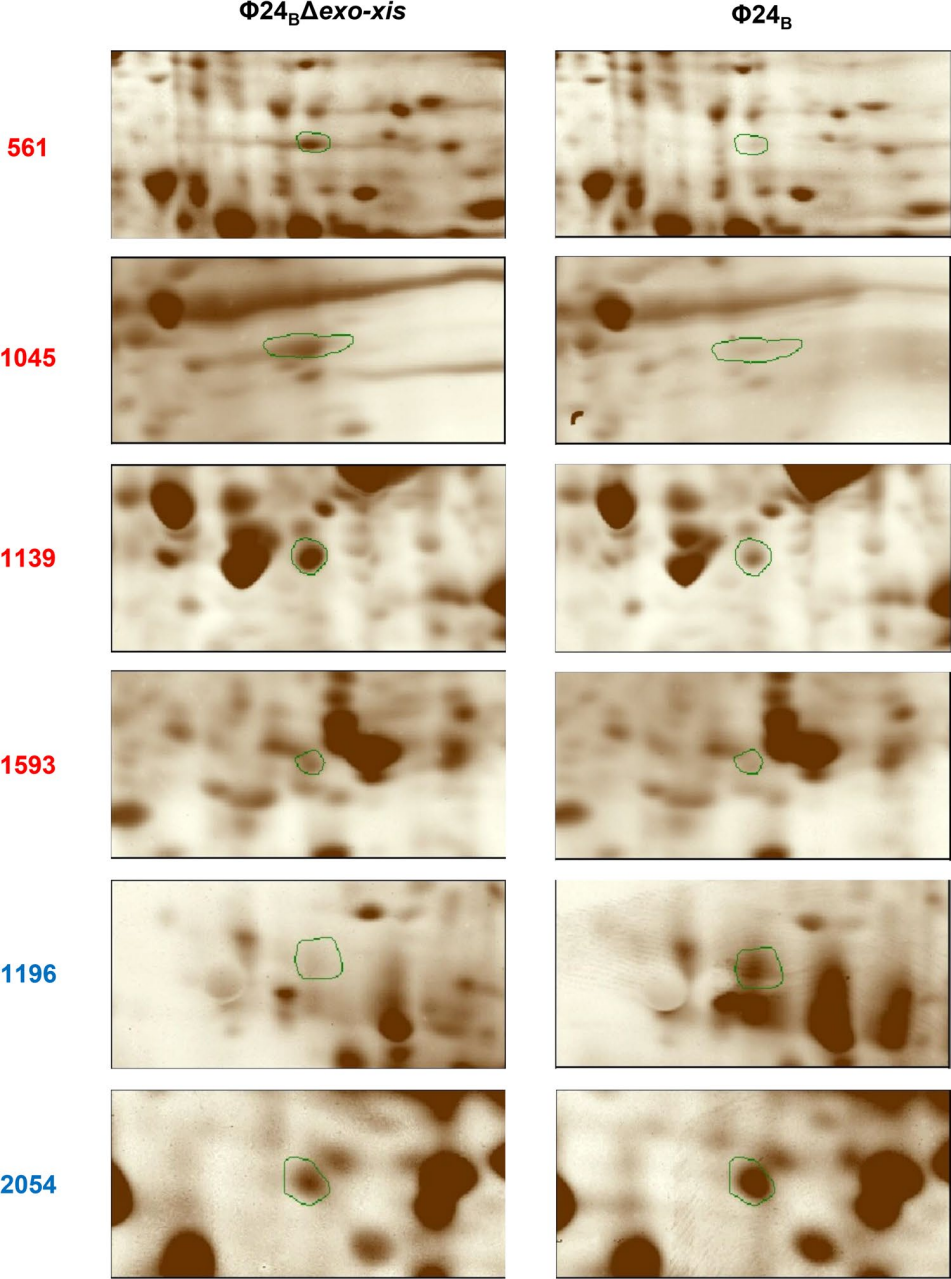
Proteins occurring at different levels in *E. coli* MG1655 infected phage Φ24_B_Δ*exo-xis* and the wild-type phage Φ24_B_. Enlarged images of 2D-PAGE fragments show a significant increase (red) or decreases (blue) in protein levels between the cells infected with investigated bacteriophages at time 30 min after infection. Protein spots of note are indicated with green line circles

Levels of proteins in *E. coli* cells infected with either Φ24_B_ or Φ24_B_Δ*exo-xis* were examined in classical proteomic experiments. Significant changes in intensities of specific spots after two-dimensional gel electrophoresis were evident as summarized in Fig. 7 and Table S2, and exemplified in Fig. 8. Identification of proteins included in specific spots visualized in Fig. 8 and their quantitative analyses are shown in Table 5.

**Table 5:** Identified proteins encoded by *E. coli*

Spot number	Protein^a^	Effectct	Accesion number	Mass (kDa)	Score^b^	Matched^c^	Function
561	Lysine-tRNA ligase	↑	gi|487586222	57.652	413	23	Lysine-tRNA ligase activity
	Elongation factor Tu, partial	↑	gi|226903110	33.78	102	5	Translation elongation factor activity
	Chaperonin GroEL	↑	gi|723112667	57.536	81	4	ATP hydrolysis activity
	Pyruvate dehydrogenase, partial	↑	gi|692966869	48.783	70	3	Pyruvate dehydrogenase (acetyl-transferring) activity
	Pyruvate kinase I	↑	gi|147276	50.773	68	2	Kinase activity
	Type I restriction-modification system methyltransferase	↑	gi|446985945	59.708	56	2	N-methyltransferase activity
1045	Maltose/maltodextrin ABC transporter ATP-binding protein MalK	↑	gi|654667397	41.179	792	64	ABC-type transporter activity
	Elongation factor Tu	↑	gi|26110363	45.035	473	28	Translation and modification
	Acriflavine resistance protein A precursor	↑	gi|26106874	43.566	396	12	Transmembrane transporter activity
	TolB protein, chain A	↑	gi|12084602	47.123	378	19	An essential component of the Tol-dependent translocation system and its interactions with the translocation domain of colicin E9
	IctD	↑	gi|466743	42.954	161	7	L-lactate dehydrogenase activity
	ORF_f388	↑	gi|606022	43.894	89	3	23S rRNA (guanine(1835)-N(2))-methyltransferase RlmG
	Cysteine desulfurase	↑	gi|723053185	45.248	87	5	Hydrolase activity
	Pectinesterase	↑	gi|192926752	46.283	87	3	Pectinesterase activity
1139	Maltodextrin-binding protein, chain A	↑	gi|13787059	40.625	1242	79	Carbohydrate transmembrane transporter activity
	Rod shape-determining protein MreB	↑	gi|485737745	37.127	498	34	ATP binding
	UTP-glucose-1-phosphate uridylyltransferase	↑	gi|377963908	32.806	154	8	UTP:glucose-1-phosphate uridylyltransferase activity
	Succinyl-diaminopimelate desuccinylase	↑	gi|447200557	41.584	135	9	Succinyl-diaminopimelate desuccinylase activity
	Aspartate-semialdehyde dehydrogenase	↑	gi|26110473	42.666	130	9	Amino acid biosynthesis: lysine
	Class II fructose-bisphosphatase	↑	gi|447173381	36.024	107	4	Fructose 1,6-bisphosphate 1-phosphatase activity
	Maltose/maltodextrin ABC transporter ATP-binding protein MalK	↑	gi|654667397	41.179	82	3	ABC-type transporter activity
	RecA protein, partial	↑	gi|512123927	42.315	70	3	DNA repair
1593	Outer membrane protein A, partial	↑	gi|558736244	25.901	314	12	Porin activity
	Triose-phosphate isomerase	↑	gi|486185246	27.338	259	14	Triose-phosphate isomerase activity
	NADH dehydrogenase I, chain B	↑	gi|25298992	25.337	195	8	Oxidoreductase activity
	Maltodextrin binding protein, chain 1	↑	gi|809263	39.897	116	4	Maltose/maltodextrin-binding
	Peptidase E	↑	gi|431248598	12.597	86	4	Dipeptidase and serine-type peptidase activity
1196	Outer membrane porin F, chain A	↓	gi|6729727	37.018	107	6	Porin activity
2054	ORF_f118	↓	gi|882488	14.113	175	9	Unknown function
	50S ribosomal protein L21	↓	gi|446193546	11.557	143	5	Structural constituent of ribosome
	Probable formate acetyltransferase yfiD	↓	gi|25285925	14.283	83	4	Catalytic activity

Up arrows indicate increases or down decreases in level proteins after infection of host bacteria with phage Φ24_B_Δ*exo-xis* relative to that of wild-type phage Φ24_B_

^a^Protein assignment based on LC-MS/MS identification

^b^Mascot score

^c^Number of peptides matched in MS analysis

These analyses confirmed that the *exo-xis* region has significant effects on expression of genes in the phage-infected host cells at the level of gene products. The proteins which levels were affected by this region included, but were not limited to, those involved in the development of lambdoid bacteriophages, like molecular chaperones, type I restriction-modification system methyltransferase, RecA, and peptidase E. Modulation of levels of the RecA protein by the *exo-xis* region might be of particular interest, as this protein is crucial for induction of lambdoid prophages (for a review, see Węgrzyn et al. 2012). Upon binding to single-stranded DNA regions (often appearing as a result of DNA damage), RecA is transformed to its active form, called RecA*, which causes the autocleavage of the phage-encoded cI repressor. This results in de-repression of phage promoters, prophage induction, and subsequent lytic development (Węgrzyn et al. 2012). Therefore, these results corroborate the proposal that the *exo-xis* region has a role in the regulation of phage development in response to various environmental conditions. Similarly, changes in levels of molecular chaperones in response to the presence/absence of the *exo-xis* region further support the above hypothesis on the biological role of this phage genome region.

### Metabolomic analyses of the *exo*-*xis* effects on levels of metabolites in host cells

**Fig. 9:**
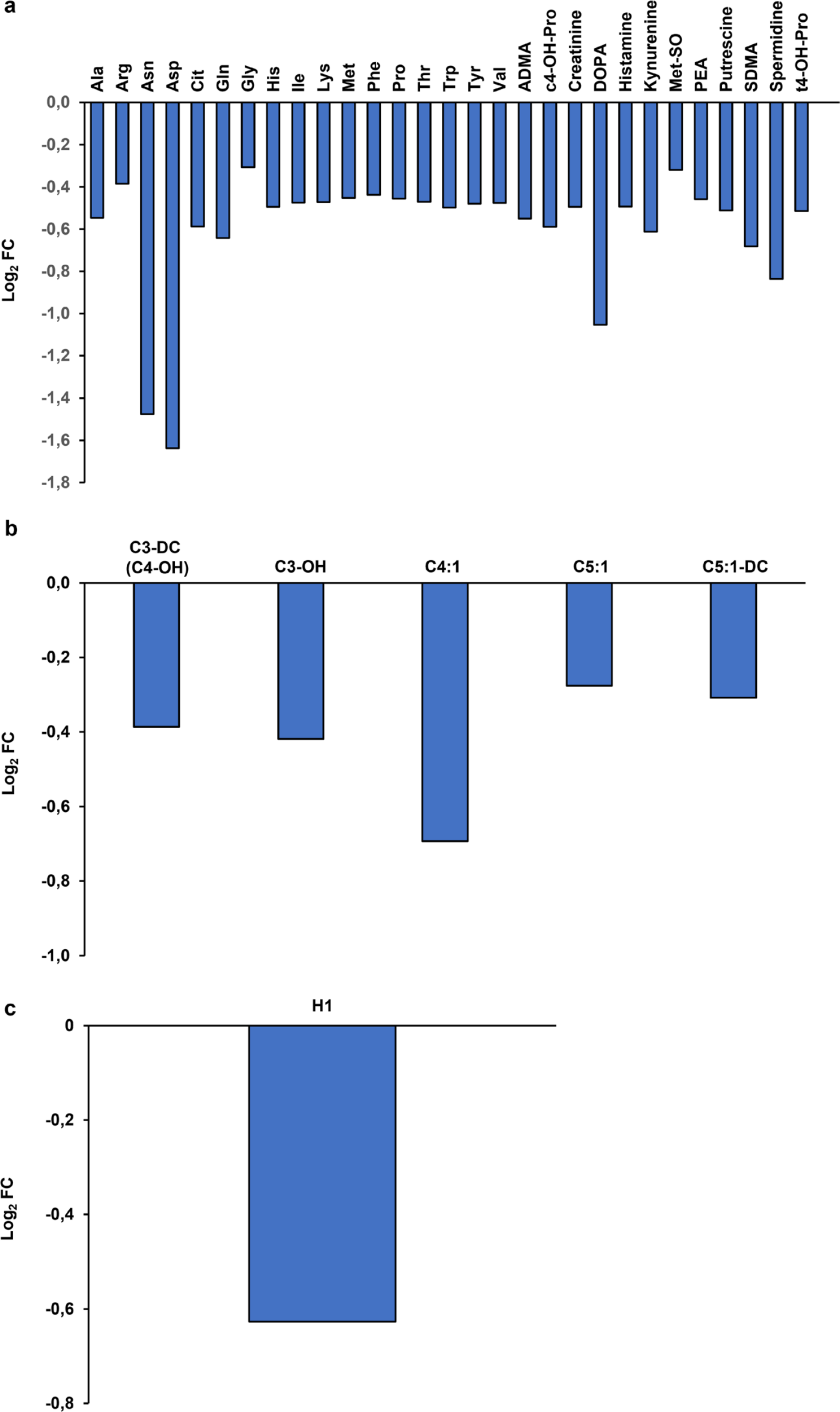
Changes in the levels of bacterial metabolites in *E. coli* MG1655 infected with phage Φ24_B_Δ*exo-xis* relative to host bacteria infected with wild-type phage Φ24_B_ at time 30 min after infection. Analyses of amino acids and biogenic amines (**a**), acylcarnitines (**b**), and hexoses (**c**) are demonstrated

To test effects of the *exo-xis* region on the cellular metabolism of host cells infected with either Φ24_B_ or Φ24_B_Δ*exo-xis*, metabolomic analyses were performed (Table S3). The most striking difference between Φ24_B_- and Φ24_B_Δ*exo-xis*-infected *E. coli* was significantly lowered levels of many amin acids and several biogenic amines in the latter host (Fig. 9a). This strongly suggest that the *exo-xis* region is required to maintain production of amino acids in infected cells which should facilitate effective synthesis of phage-encoded proteins during the lytic development. Interestingly, levels of acylcarnitines and (Fig. 9b) and hexoses (Fig. 9c) were also decreased in Φ24_B_Δ*exo-xis*-infected cells relative to those infected with the wild-type phage.

The results of metabolomic analyses suggest that the *exo-xis* region of Φ24_B_ supports the bacteriophage lytic development also by stimulating expression of a battery of genes involved in production of compounds which should support effective production of progeny virions, like amino acids and hexoses. This might be another mechanism by which genes included in the investigated region of the Φ24_B_ phage ensure efficient lytic development of this virus.

## Discussion

Previous studies suggested that genes from the *exo-xis* region of lambdoid bacteriophage genome may influence lysogenization and prophage induction processes (Bloch et al. 2013, 2014). Further studies led to the hypothesis that proteins encoded in the *exo-xis* region play roles in transcription, possibly through regulating RNA polymerase (Kwan et al. 2013; Dydecka et al. 2017, 2018, 2020; Zdrojewska et al. 2019; Tong et al. 2020; Donaldson 2021). In this light, we investigated global effects of the *exo-xis* region on gene expression and metabolism in *E. coli* cells infected with the Stx phage Φ24_B_.

Experiments with the Φ24_B_ bacteriophage devoid of the *exo-xis* region indicated that the mutant virus preferred lysogenization rather than the lytic development (Fig. 2). These results corroborate the proposal that genes included in this region are important for replication of bacteriophages and production of phage progeny. Interestingly, mutant phages were less effective in adsorption to the host cells, suggesting that the *exo-xis* region ensures proper assembly of the progeny virions. Although the mechanism of this phenomenon remains to be elucidated, transcriptomic and proteomic studies, performed in this work, provide an interesting possibility. Namely, expression of genes coding for molecular chaperones (DnaK, DnaJ, GrpE, GroELS) was down-regulated in the Φ24_B_Δ*exo-xis*-infected cells relative to those infected with the wild-type virus. Since chaperones (especially GroELS) are required to assembly phage virions properly (Węgrzyn et al. 2012), their deficit might negatively influence this process, resulting in production of partially defective viral particles. Intriguingly, it was demonstrated that Stx phages are significantly more sensitive to UV irradiation than bacteriophage λ which arose from formation of less stable virions (Bloch et al. 2015). Whether this phenomenon is influenced by the *exo-xis* functions remains to be tested.

The general conclusion from the transcriptomic, proteomic, and metabolomic studies performed in this work is that the *exo-xis* region facilitates lytic development of the Φ24_B_ bacteriophage by regulating expression of a battery of phage and host genes involved in the lysis vs. lysogenization decision (especially phage genes *c*I, *c*II, and *N*), coding for molecular chaperones, and required for metabolism of amino acids, hexoses, and some other compounds, like biogenic amines and acyl carnitines. This corroborates recent predictions about the regulation of gene expression by products of genes included in the *exo-xis* region, based on use of the AlphaFold and RoseTTAFold machine learning algorithms (Donaldson 2021).

The effects of bacteriophage genes or their products on expression of host genes have been reported previously, also for Stx bacteriophages, though such works were not numerous. Microarray analyses of non-lysogenic *E. coli* and those lysogenic by the Stx phage ΦMin27 indicated that prophage-located genes caused changes in expression of various host genes, leading to enhanced host resistance to low-pH conditions and to more effective motility (Su et al. 2010). Similar results were also obtained in studies with the Φ24_B_ bacteriophage, where transcriptomic analyses identified enhanced expression of the *gad* operon, involved in the bacteria acid resistance (Veses-Garcia et al. 2015). More recent transcriptomic studies with hosts bearing different Stx prophages (either ϕO104 or ϕPA8) demonstrated significant changes in expression of *E. coli* genes encoding enzymes involved in source utilization (Berger et al. 2019). Specifically, lysogenic cells revealed enhanced expression of genes which products are involved in mixed acid fermentation, whereas expression of those encoding enzymes of the Krebs cycle, NADH dehydrogenase I, and polypeptides required for the transport and assimilation of carbon sources was impaired, relative to non-lysogenic *E. coli* (Berger et al. 2019). Hence, it appears that the presence of the Stx prophage causes inhibition of aerobic metabolism while activation of mixed acid fermentation.

Interestingly, the *gad* operon expression was demonstrated in this work to be modulated by the *exo-xis* region of this phage (Fig. 5). Moreover, another operon which expression was found to be influenced by *exo-xis* is *nrf* (Fig. 5). Expression of this operon enhances bacterial growth under anaerobic conditions, and a promoter of this operon has been demonstrated previously to be more active when *E. coli* is lysogenic with an Stx phage (Godfrey et al. 2017). Although, to our knowledge, effects of Stx phages on host metabolome were not demonstrated previously, it is worth noting that phage-specific metabolic alterations were reported for six different bacteriophages infecting *Pseudomonas aeruginosa* (De Smet et al. 2016).

In conclusion, the *exo-xis* region of the Φ24_B_ bacteriophage genome supports lytic development of the bacteriophage over lysogenization through significantly modulating expression of a battery of phage and host genes. The phage-mediated changes include changes in levels of molecular chaperones, proteins involved in acid tolerance by the host cells, and proteins facilitating *E. coli* growth under anaerobic conditions. Moreover, the *exo-xis* region is required for maintaining relatively high levels of amino acids, biogenic amines, acylcarnitines, and hexoses. Therefore, the *exo-xis* region, playing an important role in the lysis vs. lysogenization decision and the phage lytic development, might be considered as a potential target for anti-STEC/EHEC drugs, as impairment of the propagation of Stx phages should reduce virulence of the *E. coli* strains producing Shiga toxins due to inhibition of expression of genes coding for these toxins and located in genomes of these phages.

### Supplementary information

ESM 1 (XLSX 232 kb)

ESM 2 (XLSX 476 kb)

ESM 3 (XLSX 41 kb)
